# LONELINESS AND SUICIDE IDEATION IN OLDER ADULTS: A LONGITUDINAL INVESTIGATION

**DOI:** 10.1093/geroni/igz038.2205

**Published:** 2019-11-08

**Authors:** Marnin J Heisel

**Affiliations:** The University of Western Ontario, London, Ontario, Canada

## Abstract

Older adults have the highest rates of suicide globally, necessitating theory and research investigating suicide and its prevention in later-life. The experience of loneliness is significantly associated with depression, hopelessness, negative health outcomes, and mortality among older adults. Yet, relatively little research has focused on the role of loneliness in conferring suicide risk in later life. The purpose of the present study was thus to investigate the potential associations between loneliness and suicide ideation and behavior in a sample of community-residing older adults recruited into a larger two-year longitudinal study of psychological risk and resiliency to later-life suicide ideation. We specifically recruited 173 adults, 65 years or older, from community locations in a medium-sized Canadian city, for a study on “healthy aging.” Participants completed measures of positive and negative psychological variables, including depression, loneliness, and suicide ideation at a baseline assessment, and again at 2-4 week, 6-12 month, and 1-2 year follow-up points. Findings indicated that loneliness (UCLA Loneliness Scale) was significantly positively associated with concurrent depression and suicide ideation, negatively associated with psychological well-being and perceived social support, and differentiated between participants who endorsed or denied having ever engaged in suicide behavior. Baseline loneliness also explained significant variability in the onset of suicide ideation over a 1-2 year period of follow-up, controlling for age, sex, and baseline depression and suicide ideation. These findings will be discussed in the context of the need for increased focus on psychosocial factors when assessing and intervening to reduce suicide risk in older adults.

